# Dynamics of Breast Cancer under Different Rates of Chemoradiotherapy

**DOI:** 10.1155/2019/5216346

**Published:** 2019-09-11

**Authors:** Sara B. Mkango, Nyimvua Shaban, Eunice Mureithi, Twalib Ngoma

**Affiliations:** ^1^Department of Mathematics, University of Dar es Salaam, P.O. Box 35062, Dar es Salaam, Tanzania; ^2^Department of Clinical Oncology, Muhimbili University of Health and Allied Sciences, P.O. Box 65000, Dar es Salaam, Tanzania

## Abstract

A type of cancer which originates from the breast tissue is referred to as breast cancer. Globally, it is the most common cause of death in women. Treatments such as radiotherapy, chemotherapy, hormone therapy, immunotherapy, and gene therapy are the main strategies in the fight against breast cancer. The present study aims at investigating the effects of the combined radiotherapy and chemotherapy as a way to treat breast cancer, and different treatment approaches are incorporated into the model. Also, the model is fitted to data on patients with breast cancer in Tanzania. We determine new treatment strategies, and finally, we show that when sufficient amount of chemotherapy and radiotherapy with a low decay rate is used, the drug will be significantly more effective in combating the disease while health cells remain above the threshold.

## 1. Introduction

Cancer begins when healthy cells in the breast change and grow out of control, forming a mass or sheet of cells called tumor. Usually breast cancer occurs either in the inner lining of milk ducts, known as ductal carcinomas, or the lobules of the breast, known as lobular carcinomas. Breast cancer occurs in humans and other mammals. While an overwhelming majority of human cases occur in women, breast cancer occurs in men as well. Globally, it is the most common cause of death in women [1–3]. Out of approximately 8.6 million women diagnosed with cancer in 2018, 2.1 million were breast cancer cases and 57% of the 2.1 million were from developing countries. During the same year, 626,679 breast cancer deaths were recorded, majority of which were from sub-Saharan African countries [1, 4].

Studies show that the incidence of breast cancer in sub-Saharan African countries is increasing, and this concurs with the World Health Organization (WHO) report in 2015. It is estimated that, by 2025, over 19.3 million women, predominantly from sub-Saharan African countries, will be suffering from breast cancer. The highest prevalence rate is noted to be in East, North, and West Africa [4]. In Tanzania, for instance, breast cancer represents 14.4% of new cancers among women. The age-standardized breast cancer incidence in Tanzania is 19.4 per 100,000, and the age-standardized breast cancer mortality rate is 9.7 per 100,000. This means mortality-to-incidence ratio is 0.5, which indicates that half of all women diagnosed with breast cancer in Tanzania will die of the disease. The number of new breast cancer cases is projected to increase from 2,732 in 2012, to 4,961 cases in 2030, an increase of 82%. Projections for breast cancer deaths follow the same pattern, with an increase of 80% in breast cancer deaths by 2030 [5, 6].

Many strategies have been used to control this disease from the populations, for instance, prevention, early detection, diagnosis, and treatment. Although prevention and early detection have been the cornerstone of breast cancer control in low- and middle-income countries, treatment has remained the main strategy in the fight against breast cancer [7, 8]. For example, in Tanzania, the struggle to combat breast cancer is being led by Medical Women Associations of Tanzania (MEWATA) and Tanzania Breast Health Care Assessment (TBHCA). For successful control of breast cancer, treatment should be administered so as to control the growth of breast tumor cells.

One of the purposes of modelling the dynamics of breast cancer disease is to provide a rational basis for policy design to control the spread of cancer cells. Mathematical models such as in [9–11] have been used to study some of the interactions between tumor and immune system, tumor-immune system with treatment, and tumor growth based on tumor population dynamics. For instance, the model developed in [12] considered the interaction between transforming growth factor- (TGF-)inhibitor and vaccine treatments. They have showed that vaccine alone allows for the development of a significant and long-term immune response that is minimally affected by the TGF that is present at later time points and the TGF-inhibitor alone provides conditions that help the populations of immune cells to expand during the initial phases of tumor presentation.

The model developed in [13] specifically considers the effects of the cytokine interleukin-2 (IL-2). Their results indicate that IL-2 treatment alone does not boost the immune system enough to clear the tumor. However, large amounts can have pathologic effects, but the combined effects of IL-2 and adoptive cellular immunotherapy (ACI) showed to be the best options for the clearance of tumor. In this work, we consider the stage at which cancer cells fail to be controlled even if the immune system is boosted, that is, invasive breast cancer that spreads to nearby tissue. Tumor-immune interactions were also studied in [14], where the authors considered a patient suffering from brain tumor and formulate a mathematical model for immunotherapy with T11 target structure (T11TS). The qualitative results presented in their work showed that, without T11 target structure, the body's own defence mechanism fails to control the growth of malignant glioma cells, while with T11 target structure, there is significant decrease in the cell count of malignant glioma cells. They suggested that T11 target structure needs to be investigated in human. In the work of [15], a mathematical model governing cancer growth on a cell population level with combination of immune, vaccine, and chemotherapy treatments was investigated. It was found that neither chemotherapy nor immunotherapy alone is sufficient to control tumor growth, but in combination, the therapies are able to eliminate the entire tumor. There is a lot of literature that addresses the development of various mathematical models of cancer and treatment, for example, [16–26]. The work in [27] demonstrated the crucial role played by the immune system in the process of tumor elimination. However, the results showed that despite immune pressure, cancer is able to persist if the cells are able to mutate fast and the immune response is not strong enough.

Despite the overall success of these mathematical models, it is evident from all literatures presented that most mathematical models describe tumor-immune system interaction based on cancer in general. Since different cancers respond differently to treatment, the goal of this study is to focus on a specific cancer (that is breast cancer) rather than modelling disease in general. This goal goes in line with the suggestion from [20]. Based on old and recent existing models such as [15, 23, 28–31], we develop a mathematical model that captures the effects of treatment on the dynamics of breast cancer. The use of combination therapy, such as chemotherapy and radiotherapy, has not been investigated to study the dynamics of breast cancer disease. This is important because radiotherapy and low-dose chemotherapy after surgery help destroy any remaining cancer cells [32]. In order to better understand the dynamics of the disease, we consider three treatment approaches: single therapy, combination therapy, and amount of drug doses administered while reducing the side effects. The outline of this work is organized as follows: Materials and Methods are presented in Section 2. Results and discussion are presented in Section 3. Finally, we conclude and give the remarks in Section 4.

## 2. Materials and Methods

### 2.1. The Model

We develop a model by assuming the logistic growth of cell populations in the absence of chemotherapy and radiotherapy. Some tumor cells are assumed to avoid immune response control due to succession of mutations leading to the development of immune-resistant cells [27]. At any time *t*, we consider immune response as natural killer cells denoted by (*I*(*t*)) and describe its dynamics by assuming that the source of *I*(*t*) is constantly infused in the body daily. The model views the tumor as a single compartmental population and divide it into two types of cell subpopulations, namely, the tumor-sensitive cells denoted as *T*(*t*) and the resistant cells, *T*_*R*_(*t*). Since the issue at hand is invasive breast cancer that spreads to nearby tissue, it is assumed that the impact of normal cells on the tumor cells is negligible. The model equations are given below.  Table 1 gives explanations of the terms.

The dynamic of tumor-sensitive cells is represented by

(1) $$\begin{array}{c} \frac{dT}{dt}=r_{1}T\left(1-\frac{T}{T_{\max}}\right)-\alpha_{1}IT-\mu T-a_{T}\left(1-e^{-\delta_{1}M}\right)T-b_{T}\left(1-e^{-\delta_{2}R}\right)T, \end{array}$$

where we adapt the following terms, that is, growth rate, natural killer induced tumor death, mutation rate, and death of tumor-sensitive cells due to chemotherapy from [23, 29]. We added and assumed the death of tumor-sensitive cells due to radiotherapy is the same as chemotherapy.

The dynamics of tumor-resistant cells, *T*_*R*_(*t*), is represented by

(2) $$\begin{array}{c} \frac{dT_{R}}{dt}=r_{2}T_{R}\left(1-\frac{T_{R}}{T_{\max}}\right)+\mu T-a_{T_{R}}\left(1-e^{-\delta_{1}M}\right)T_{R}-b_{T_{R}}\left(1-e^{-\delta_{2}R}\right)T_{R}, \end{array}$$

where we assumed that tumor-resistant cells grow logistically and the second term on the right-hand side of (2) is adapted as in [23]. The death of tumor-sensitive cells due to chemotherapy and radiotherapy are assumed to be the same as in equation (1).

The normal cell compartment is represented by *N*(*t*) and the dynamics, as adapted from [29], is represented by using the following equation:

(3) $$\begin{array}{c} \frac{dN}{dt}=r_{3}N\left(1-\frac{N}{N_{\max}}\right)+kT\left(1-\frac{T}{T^{*}}\right)-a_{N}\left(1-e^{-\delta_{1}M}\right)N-b_{N}\left(1-e^{-\delta_{2}R}\right)N. \end{array}$$

Since the issue here is invasive breast cancer, the competition between tumor cells and normal cells, for resources like nutrients, oxygen, and environment in a small volume, is not significant. We add the second term by assuming that normal cells are activated by the presence of tumor cells and the ability of tumor cells to inhibit the normal cells growth increases as the population of tumor cells passes the critical value, i.e., *T* > *T*^*∗*^. Explanations for the last two terms are similar to equation (1), and except here, we consider *N*(*t*).

Next we consider the immune cells, *I*(*t*), as adapted from [28, 29, 31], and the dynamics is represented by using the following equation:

(4) $$\begin{array}{c} \frac{dI}{dt}=s+\frac{\varepsilon_{1}IT}{\varepsilon_{2}+T}-dI-\alpha_{2}IT-a_{I}\left(1-e^{-\delta_{1}M}\right)I-b_{I}\left(1-e^{-\delta_{2}R}\right)I, \end{array}$$

where we added the last two terms as explained in equation (1).

We also consider the concentration of chemotherapy and radiotherapy denoted by *M*(*t*) and *R*(*t*), respectively. To control breast cancer progression, an equation with chemotherapy treatment is included as adapted in [15, 23, 29, 30]. The interaction between chemotherapy and all cells is found to follow an exponential saturation kinetics model, and this saturation have been validated by [33] for a reasonable number of chemotherapeutic drugs. Since the model considered in this study includes chemotherapy and radiotherapy, the later also is assumed to interact in a similar way. In the same way as in [31, 34, 35], the present model ignores any spatial dependence of the dynamics. The dynamics of chemotherapy and radiotherapy, respectively, are represented by using the following equations:

(5) $$\begin{array}{c} \frac{dM}{dt}=V_{M}\left(t\right)-d_{1}M,\\ \frac{dR}{dt}=V_{R}\left(t\right)-d_{2}R. \end{array}$$

Thus, the developed model with chemotherapy and radiotherapy treatment is composed of six ordinary differential equations as follows:

(6) $$\begin{array}{c} \frac{dT}{dt}=r_{1}T\left(1-\frac{T}{T_{\max}}\right)-\alpha_{1}IT-\mu T-a_{T}\left(1-e^{-\delta_{1}M}\right)T-b_{T}\left(1-e^{-\delta_{2}R}\right)T,\\ \frac{dT_{R}}{dt}=r_{2}T_{R}\left(1-\frac{T_{R}}{T_{\max}}\right)+\mu T-a_{T_{R}}\left(1-e^{-\delta_{1}M}\right)T_{R}-b_{T_{R}}\left(1-e^{-\delta_{2}R}\right)T_{R},\\ \frac{dN}{dt}=r_{3}N\left(1-\frac{N}{N_{\max}}\right)+kT\left(1-\frac{T}{T^{*}}\right)-a_{N}\left(1-e^{-\delta_{1}M}\right)N-b_{N}\left(1-e^{-\delta_{2}R}\right)N,\\ \frac{dI}{dt}=s+\frac{\varepsilon_{1}IT}{\varepsilon_{2}+T}-dI-\alpha_{2}IT-a_{I}\left(1-e^{-\delta_{1}M}\right)I-b_{I}\left(1-e^{-\delta_{2}R}\right)I,\\ \frac{dM}{dt}=V_{M}\left(t\right)-d_{1}M,\\ \frac{dR}{dt}=V_{R}\left(t\right)-d_{2}R. \end{array}$$

The initial conditions are *T*(0)=*T*_*o*_ ≥ 0, *T*_*R*_(0)=*T*_*R*_*o*__ ≥ 0, *N*(0)=*N*_*o*_ > 0, *I*(0)=*I*_*o*_ > 0, *M*(0)=*M*_*o*_ ≥ 0, and *R*(0)=*R*_*o*_ ≥ 0.

#### 2.1.1. The Parameters

Determination of parameters is very important for a complete model. Tables 2 and 3 provide quick references for the parameter values and their description used in our model.

#### 2.1.2. Equilibrium States

We obtained reasonable equilibrium points for the tumor-free condition and for the endemic condition. We first consider the case of a tumor-free condition, where normal cells and immune cells exist, using the parameter values given in Table 3 except *r*_1_ = 0.00431 and *r*_2_ = 0.0025. That is, *N*^•^ = 2.7428 × 10^7^ and *I*^•^ = 1.3793 × 10^6^, and tumor cell populations decline to zero (*T*^•^ = *T*_*R*_^•^ ≈ 0), where (*N*^•^, *I*^•^, *T*^•^,  and *T*_*R*_^•^) represents cell at tumor-free equilibrium.

Here,

- *r*_3_ > 0
- *I*^•^ > (*r*_1_/*α*_1_)

which shows that tumor-free condition is locally asymptotically stable.

Next, we consider the case of a coexisting equilibrium using the parameter values given in Table 3 except *r*_3_=0.007. Using these parameter values, tumor-sensitive cells, tumor-resistant cells, normal cells, immune cells, the amount (or concentration) of chemotherapeutic drug, and the amount (or concentration) of radiotherapy all exist. That is, *T*^*⊛*^=4.0558 × 10^7^, *T*_*R*_^*⊛*^=3.1085 × 10^7^, *N*^*⊛*^=4.0348 × 10^6^, *I*^*⊛*^=2.1704 × 10^5^, *M*^*⊛*^=44.4873, and *R*^*⊛*^=44.4873, where (*T*^*⊛*^,  *T*_*R*_^*⊛*^,  *N*^*⊛*^,  *I*^*⊛*^,  *M*^*⊛*^,  *R*^*⊛*^) represents cells at the endemic state.

This indicates that the endemic equilibrium point is locally asymptotically stable.

Here, *I*^*⊛*^ < (*r*_1_/*α*1) − ((*a*_*T*_(1 − *e*^−*M*^*⊛*^^)+*b*_*T*_(1 − *e*^−*R*^*⊛*^^))/*α*_1_) which shows that the tumor-free equilibrium becomes unstable and only the coexisting equilibrium exists. In other words, through this result, we see that any tumor size *T* > 0 will grow to this maximal tumor size. If the tumor is not reduced, then the immune cell population cannot sustain itself. Therefore, biologically this situation means that the immune system begins to fail. Note that, since we are dealing with the population of cells, we consider only positive populations for *N*^•^ and *T*_*R*_^•^.

### 2.2. Data Fitting

As an example, in order to check model conformity with the real data, we fit the model system (6) with only two data points obtained in the form of recent reported cases (of one patient) of breast cancer tumor volume before and during treatment in Tanzania. We use parameter ranges selected from some published literatures and others are assumed. Tables 2 and 3, respectively, give descriptions and estimated parameter ranges.

Before treatment, the data were collected on September 2017 (tumor size and diameter was 2.147 cm), and during treatment, after 140 days, the data were collected on February 2018 (tumor size and diameter was approximatly 0.58 cm) from Ocean Road Cancer Institute. The estimation process of the parameter point values attempts to find the best concordance between computed and observed data. It can be carried out by trial and error or by the use of software programs designed to find parameters that give the best fit.

We used the least squares curve fitting method, where a Matlab code is used when unknown parameter values are given a lower and upper bound from which the set of parameters values that produce the best fit were obtained. The parameter ranges and resultant point values are given in Table 3. The following initial conditions have been considered in the curve fitting: *T*_*o*_=3500000, *T*_*R*_*o*__=1400000, *N*_*o*_=1000000, *I*_*o*_=1000000, *M*_*o*_=0, *R*_*o*_=0, and *T*_*T*_*o*__=4900000, where *T*_*T*_*o*__=*T*_*o*_+*T*_*R*_*o*__ represents the total breast cancer cells.

**Figure 1:**
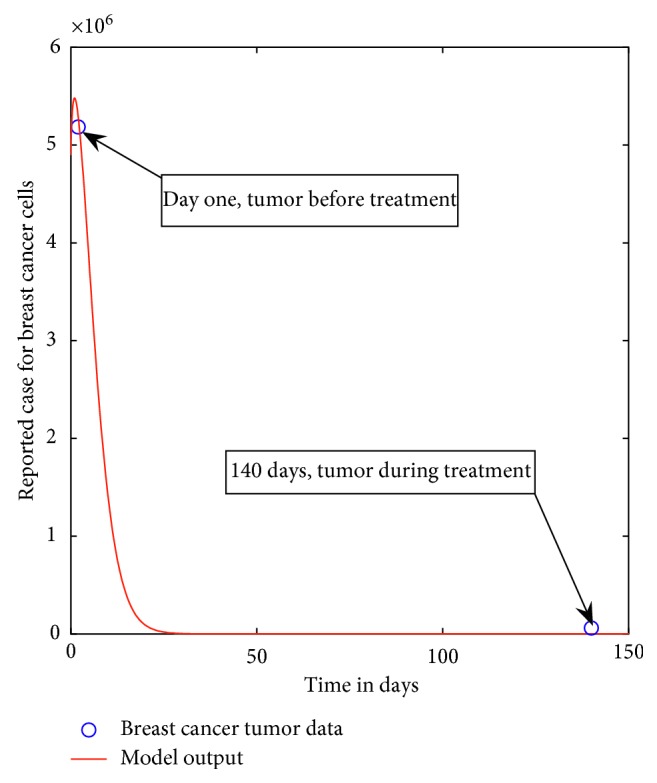
Model system (6) fitted to data for a person with breast cancer. The blue circle indicates the actual data and the solid red line indicates the model fit to the data.

Figure 1 demonstrates a good fit for the pair of data obtained when both chemotherapy and radiotherapy are used. The results are indicative of a decreasing breast cancer tumor in which there is a slightly significant increase of breast cancer tumor in a short period of time, followed by a significant decrease as time goes up. The breast cancer tumor extincts after 150 days. Although the results show a small sample from a person with breast cancer in Tanzania, it is indicative of the need to promote the use of combination of therapies and the need to promote the preventive mechanism against the occurrence of breast cancer.

## 3. Results and Discussion

In this section, we test the behavior of our model by using the parameter values shown in Table 3. We simulate three treatment strategies with the same initial conditions: the first strategy is when the chemotherapy drug dose is used alone, the second strategy is when radiotherapy drug dose is used alone, and the third strategy is the combination of the first and the second strategies. In all numerical simulations, the three dose tested low, standard, and high is as follows:

1. Low: *V*_*M*_=*V*_*R*_=0.25
2. Standard: *V*_*M*_=*V*_*R*_=0.5
3. High: *V*_*M*_=*V*_*R*_=1.

### 3.1. First Strategy: Chemotherapy Alone

**Figure 2:**
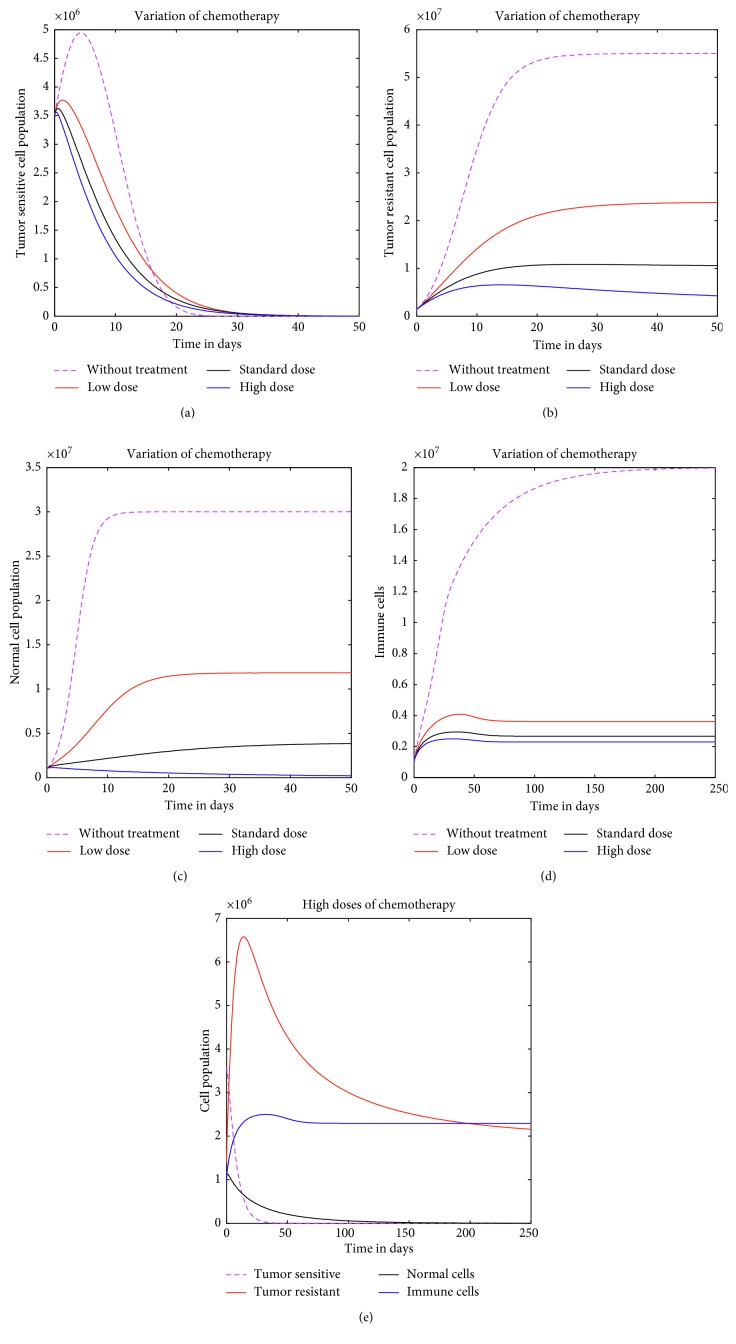
The finite continuous chemotherapeutic treatment on cell population with different cytotoxic drug doses. The inhibition time in which the tumor-sensitive cells begin to decrease is approximately *t*=20 days due to mutation rate, while the tumor-resistant cells increase to its maximum carrying capacity.

Numerical solutions with different chemotherapy doses are presented in Figure 2. Figures 2(a) and 2(b), respectively, show the evolution of tumor-sensitive cells and tumor-resistant cells, while Figures 2(c) and 2(d) show the evolution of normal cells and immune cells, respectively. The evolution of coexistence system is presented in Figure 2(e). Note that the inhibition time in which the tumor-sensitive cells begin to decrease is approximately 20 days due to mutation rate (Figure 2(a)), while the tumor-resistant cells reach the asymptote (Figure 2(b)). This indicates that tumor-resistant cells grow to the maximum carrying capacity in the host tissue in the absence of therapy. This suggests that once the tumor has been detected, there is immediate need for medical treatment. Furthermore, comparing the two figures (Figures 2(a) and 2(b)), it can be seen that as the chemotherapy drug dose increases, it reduced the volume of tumor cells but fails to eradicate the tumor-resistant cells.

The graph of immune cells (Figure 2(d)) indicates that, with high chemotherapeutic drug dose, *V*_*R*_=1, the cells drop but then remain to more than half the initial values. This is in contrast to normal cells (Figure 2(c)). In this case, we note that the normal cell population is quickly reduced to an insignificant amount compared to immune cells. Figure 2(e) shows the coexistence states with high drug dose. Here, we note that the population of tumor-resistant cell is higher compared to all population cells. This indicates that cancer cells persist even with high chemotherapy dose.

### 3.2. Second Strategy: Radiotherapy Alone

**Figure 3:**
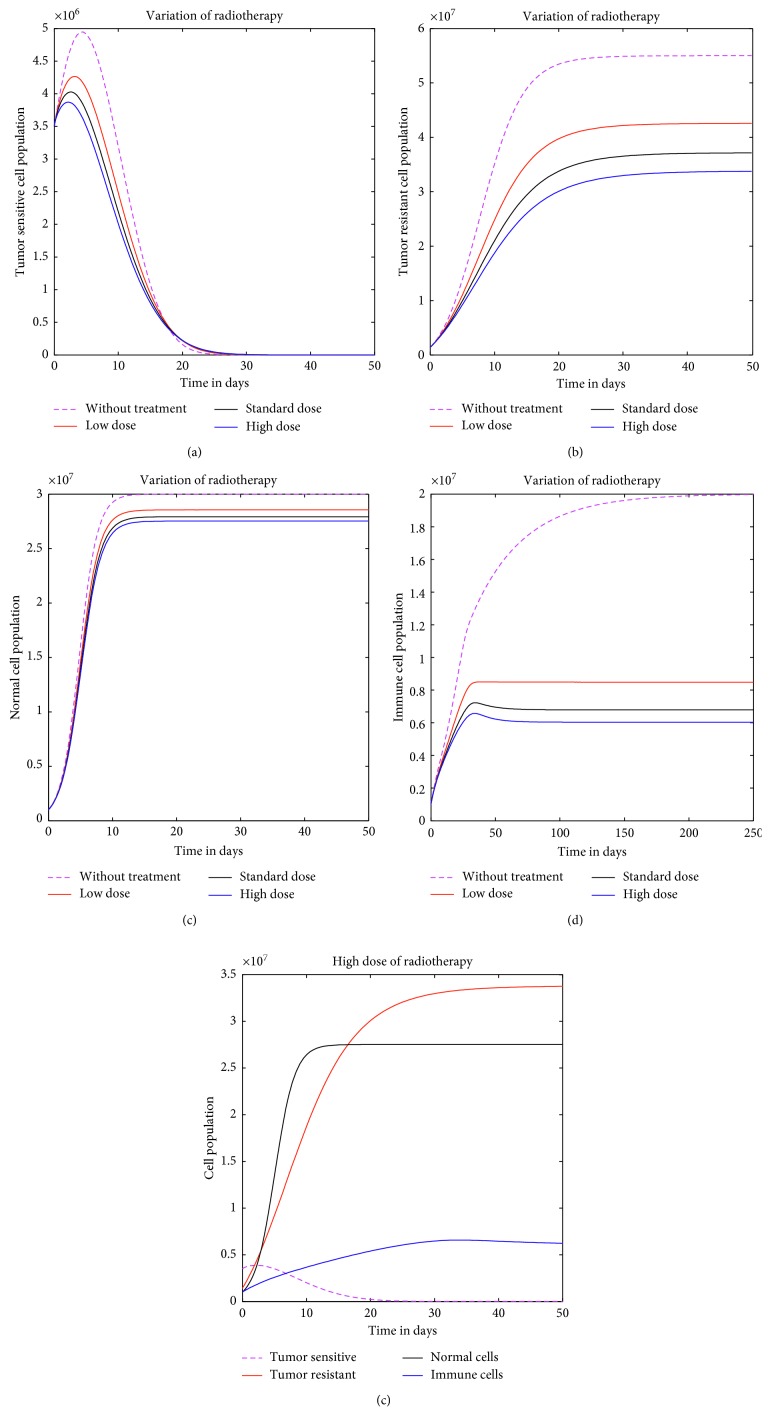
The finite continuous treatment on tumor cell population with different radiotherapy doses.

As the system of tumor-sensitive and tumor-resistant cells interact with low chemotherapy drug dose, both the population of tumor cells are highly killed (Figures 2(a) and 2(b)) compared to that when low dose of radiotherapy was used (Figures 3(a) and 3(b)). Ideal chemotherapy agents are agents that are capable of killing significant numbers of tumor cells with very high effects on the normal and immune cells populations (Figures 2(a)–2(c)). In this case, with variations of radiotherapy, the reduced number of normal cells is significant but the shrinkage of tumor volume is not significant. Hence, radiation therapy alone cannot manage to eradicate tumor cells (Figures 3(a) and 3(b)).

### 3.3. Third Strategy: Combination Therapy (Chemotherapy and Radiotherapy)

**Figure 4:**
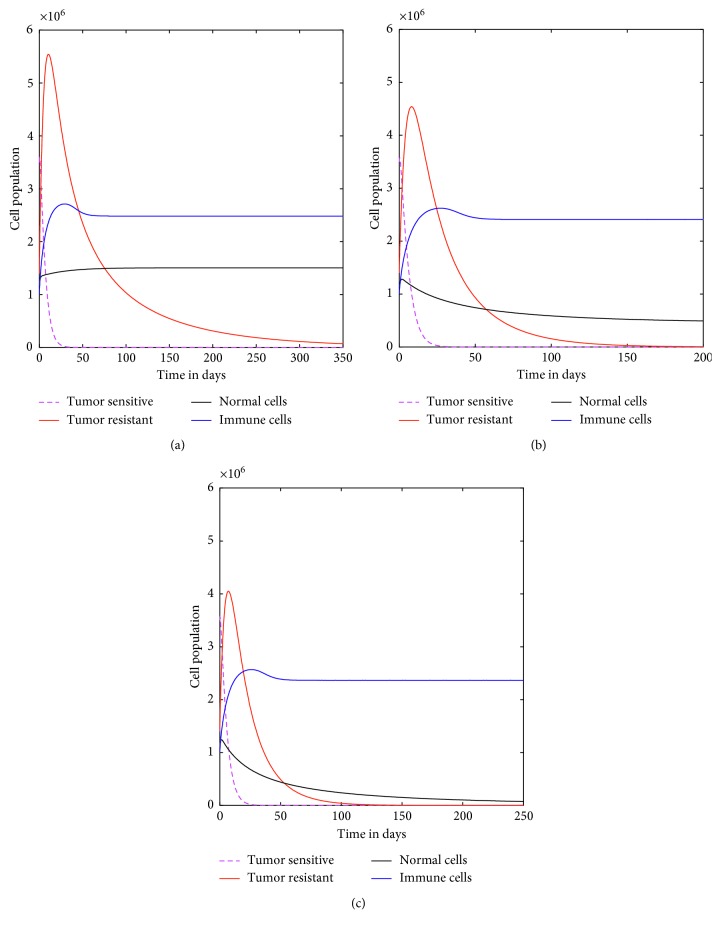
The response of the tumor subpopulations, normal cells, and immune cells to various radiotherapy doses of (a) low, (b) standard, and (c) high with standard chemotherapeutic drug dose.

Figures 4(a)–4(c)show the reduction of tumor-sensitive cells and tumor-resistant cells with combination of therapies. We keep chemotherapy in a standard dose while increasing radiotherapy drug dose. By doing that, in Figure 4(a), it can be seen that the graph of tumor-resistant cell does take longer time to reach an asymptotic horizontal value compared to Figures 4(b) and 4(c). Thus, we conclude that while eradication of tumor-sensitive cells takes a lesser period of time than that of tumor-resistant cells, there ultimately remain very few sensitive cells which accumulate mutation to become resistant cells.

It can also be seen that, from the graphs in Figure 4(a), with a low dosage of radiation, the population of tumor-resistant cells decreases but not as quickly compared to standard and higher dosage which appears to drop to zero between 100 days and 150 days. This indicates that an average infusion of radiotherapy and chemotherapy might be a vulnerable strategy to eradicate both tumor cells. In Figure 4(a), the red curve highlights the fact that tumor-resistant cells is, generally, not easy to control with low doses. That is why resistant cells have always been ascribed as a major source of failure in many therapeutic treatments [41].

Generally, insightful results are obtained when chemotherapeutic drug and radiotherapy are fixed at standard and low to standard tolerable content, per day, respectively. It is interesting to note that the healthy cells (normal cells) appear to decrease and remain above the threshold, while the natural killer cells initially increase and then drop off to its steady state under low to standard radiotherapy doses (Figures 4(a) and 4(b)).

Nevertheless, with the drug dose within the toxicity constraints, a majority of both tumor-sensitive and tumor-resistant cells are greatly reduced between 100 days and 150 days. This indicates that a low or standard infusion of radiotherapy with standard chemotherapeutic drug dose might be a valuable strategy to eradicate sensitive cells and resistant cells while keeping healthy cells above the threshold amount.

### 3.4. Numerical Sensitivity Analysis

Following the work in [42, 43], we used Latin hypercube sampling (LHS) and the partial rank correlation coefficient (PRCC) to investigate the most sensitive parameters to the model outcomes and hence to determine which of the parameters could be most effectively controlled in order to mitigate breast cancer occurrence.

**Figure 5:**
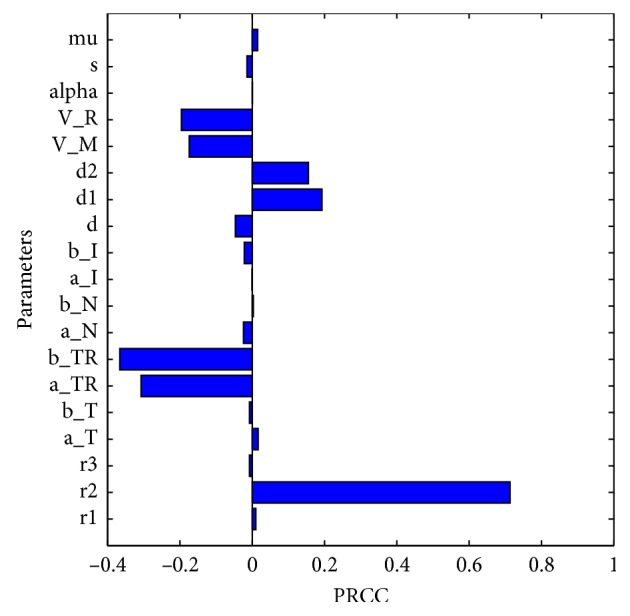
The PRCCs of model parameters with the tumor cells as the baseline variable. Table 3 shows all parameter values used. The most sensitive parameters are *r*_2_, *a*_*T*_*R*__, *b*_*T*_*R*__, *d*_1_, *d*_2_, *V*_*M*_, and *V*_*R*_.

LHS/PRCC was run and analyzed with a sample size of 100. The choice of this sample size is due to the fact that PRCC produces accurate results for a lower sample size compared to other techniques like Fourier amplitude sensitivity test (eFAST) [43].  Figure 5 displays the parameter value plotted against a bar graph of PRCCs with tumor compartment as the baseline-dependent variable. The parameters that are significantly positively correlated with tumor cells are *r*_2_, *d*_1_, and *d*_2_, while *a*_*T*_*R*__, *b*_*T*_*R*__, *V*_*M*_, and *V*_*R*_ are significantly negatively correlated.

An increase in the production of tumor-resistant cells, *r*_2_, leads to the higher number of tumor-resistant cells. Corresponding reasoning can be applied to the positive value of the PRCC for *d*_1_ and *d*_2_ indicating that when a drug with high decay rate is used, then the number of breast cancer cells increases. If the drug does not decay quickly, many breast cancer cells would be killed by the drug elimination from a surrounding tumor tissue can take place due to natural decay because chemotherapy and radiotherapy drug molecules are subject to natural decay before they are taken up by cells [25], and it is clear that if the drug does decay fast, then many drug molecules would not interact with breast cancer cells. This further suggests that if the number of breast cancer cells increases, then the external drug influx *V*_*M*_ and *V*_*R*_ should be increased. However, the increase of drug influx should be within tolerable toxicity constraints. These results appear to show that drug decay and drug influx are an important aspect to consider for chemotherapy and radiotherapy modelling.

## 4. Conclusion

A plan for the treatment of breast cancer is a key component of any overall breast cancer control plans. Its main goal is to cure breast cancer patients or prolong their life considerably, ensuring a good quality of life. Here, a deterministic model for breast cancer disease dynamics that incorporates compartment with treatment was developed.

To identify the parameter values with the highest effect on the model outcome, LHS and PRCC are used. The results show that the rate of growth of tumor-resistant cell *r*_2_ has the highest sensitivity index. This is followed by the drug decay rates (*d*_2_ and *d*_1_) for radiotherapy and chemotherapy, respectively. We have also established that the kill rate coefficients for tumor-resistant cell have relatively high negative sensitivity indexes followed by the rates of drug dose for both therapies. It is clear indication that the parameters have an impact of reducing breast cancer cells.

As demonstrated from Figures 2 and 3, the use of single therapy is not sufficient to eradicate breast cancer in either taking the standard or high drug doses of chemotherapy or radiotherapy in a model. However, we can observe that when a sufficient amount of chemotherapy and radiotherapy are used, the model system (6) is able to completely eradicate breast cancer cells (Figures 4(a) and 4(b)) while keeping health cells above the threshold. These results support the assumption that the combination of therapies increases the likelihood of eradicating cancer cells.

As with many models, the model presented here should be treated with caution because of the difficulty in the estimation of model parameters. More realistic results can be obtained if data of more than two measurements from the person with breast cancer tumor were available. Despite these shortcomings, the model still provides some useful insight into the control of breast cancer disease through the implementation of the discussed treatment strategies. Note that the same model could be used to investigate the setup of an optimal control problem relative to the model so as to minimize the number of breast tumor cells and the chemotherapeutic and radiotherapeutic doses administered.

## Figures and Tables

**Table 1:** Equation descriptions.

Equation	Term	Description	Source
*dT*/*dt*	*r*_1_ *T*(1 − (*T*/*T*_max_))	Logistic tumor-sensitive growth	[29, 30]
	−*α*_1_*IT*	NK-induced tumor death	[28, 29, 31]
	−*μT*	Tumor-sensitive cell mutation	[23]
	−*a*_*T*_(1 − *e*^−*δ*_1_*M*^)*T*	Chemotherapy-induced tumor-sensitive death	[30]
	−*a*_*T*_(1 − *e*^−*δ*_2_*R*^)*T*	Radiotherapy-induced tumor-sensitive death	Assumed

*dT*_*R*_/*dt*	*r*_2_ *T*_*R*_(1 − (*T*_*R*_/*T*_max_))	Logistic tumor-resistant growth	Assumed
	*μT*	Tumor-sensitive cell mutation	[31]
	−*a*_*T*_*R*__(1 − *e*^−*δ*_1_*M*^)*T*_*R*_	Chemotherapy-induced tumor-resistant death	Assumed
	−*b*_*T*_*R*__(1 − *e*^−*δ*_2_*R*^)*T*_*R*_	Radiotherapy-induced tumor-resistant death	Assumed

*dN*/*dT*	*r*_3_ *N*(1 − (*N*/*N*_max_))	Logistic normal growth	[23]
	*kT*(1 − (*T*/*T*^*∗*^))	Production of N cells from activated T cells	Assumed
	−*a*_*N*_(1 − *e*^−*δ*_1_*M*^)*N*	Death of normal cells due to chemotherapy toxicity	[15, 29, 30]
	−*b*_*N*_(1 − *e*^−*δ*_2_*R*^)*N*	Death of normal cells due to radiotherapy toxicity	Assumed

*dI*/*dt*	*s*	Constant source of immune cells	[28, 29, 31]
	(*ε*_1_*IT*)/(*ε*_2_+*T*)	Stimulatory effect of *T* on immune cells	[28, 29, 31]
	−*dI*	Immune turnover	[19]
	−*α*_2_*IT*	Immune death by exhaustion of tumor-killing resources	[28, 29, 31]
	−*a*_*I*_(1 − *e*^−*δ*_1_*M*^)*I*	Death of immune cells due to chemotherapy toxicity	[15, 29, 30]
	−*b*_*I*_(1 − *e*^−*δ*_2_*R*^)*I*	Death of immune cells due to radiotherapy toxicity	Assumed

*dM*/*dt*	*V*_*M*_(*t*)	Chemotherapy drug dose	[15, 29, 30]
	−*d*_1_*M*	Excretion and elimination of chemotherapy toxicity	[15, 29, 30]

*dR*/*dt*	*V*_*R*_(*t*)	Radiotherapy drug dose	Assumed
	−*d*_2_*R*	Excretion and elimination of radiotherapy toxicity	Assumed

**Table 2:** Description of parameters.

Equation	Parameter	Description
*dT*/*dt*	*r*_1_	Growth rate of tumor-sensitive cells
	*T*_max_	Carrying capacity of tumor cells
	*α*_1_	Tumor cell death rate due to immune cells
	*μ*	Mutation rate
	*a*_*T*_	Chemotherapy kill rate coefficient for tumor-sensitive cells
	*b*_*T*_	Radiotherapy kill rate coefficient for tumor-sensitive cells

*dT*_*R*_/*dt*	*r*_2_	Growth rate of tumor-resistant cells
	*a*_*T*_*R*__	Chemotherapy kill rate coefficient for tumor-resistant cells
	*b*_*T*_*R*__	Radiotherapy kill rate coefficient for tumor-resistant cells

*dN*/*dt*	*r*_3_	Growth rate of normal cells
	*N*_max_	Carrying capacity of normal cells
	*k*	Activation rate of tumor cells into normal cells
	*T* ^*∗*^	Critical size of tumor cells
	*a*_*N*_	Chemotherapy kill rate coefficient for normal cells
	*b*_*N*_	Radiotherapy kill rate coefficient for normal cells

*dI*/*dt*	*s*	Constant source of immune cells
	*d*	Natural death rate of immune cells
	*ε*_1_	Maximum immune response rate
	*ε*_2_	Steepness of immune rate
	*α*_2_	Immune cells death rate due to tumor cell response
	*a*_*I*_	Chemotherapy kill rate coefficient for immune cells
	*b*_*I*_	Radiotherapy kill rate coefficient for immune cells

*dM*/*dt*	*V*_*M*_	Chemotherapy drug dose
	*d*_1_	Chemotherapy drug decay rate

*dR*/*dt*	*V*_*R*_	Radiotherapy drug dose
	*d*_2_	Radiotherapy drug decay rate

**Table 3:** Parameters used for numerical simulation.

ODE	Parameter	Value range	Point value	Units	Source
*dT*/*dt*	*r*_1_	0.02 − 0.95	0.431	Day^−1^	[15, 31]
	*T*_max_	(1 − 6) × 10^7^	5.5 × 10^7^	Cells	Assumed
	*α*_1_	0.0 − 1.0	1 × 10^−8^	Cells day^−1^	[31]
	*μ*	0 − 0.1	0.001	Cells^−1^ day^−1^	[36, 37]
	*a*_*T*_	0.001 − 1	0.08	Day^−1^	Estimated
	*b*_*T*_	0.001 − 1	0.03	Day^−1^	Assumed

*dT*_*R*_/*dt*	*r*_2_	0.02 − 0.95	0.25	Day^−1^	Estimated
	*a*_*T*_*R*__	0.001 − 1	0.08	Day^−1^	Assumed
	*b*_*T*_*R*__	0.001 − 1	0.03	Day^−1^	Assumed

*dN*/*dt*	*r*_3_	0.02 − 0.90	0.65	Day^−1^	[31]
	*N*_max_	(1 − 5) × 10^7^	3 × 10^7^	Cells	Assumed
	*k*	0.00 − 1.00	1.1 × 10^−6^	Day^−1^	[23]
	*T* ^*∗*^	(1 − 9) × 10^5^	5 × 10^5^	Cells	[38]
	*a*_*N*_	0.001 − 1	0.03	Day^−1^	Estimated
	*b*_*N*_	0.001 − 1	0.03	Day^−1^	Assumed

*dI*/*dt*	*s*	(0.1 − 1.5)^5^	100000	Day^−1^	[31]
	*d*	0.001 − 1.0	0.0125	Day^−1^	[31, 39]
	*ε*_1_	0.0 − 1.0	0.0206	Day^−1^	[29, 31]
	*ε*_2_	50 − 50000	30000	Day^−1^	[29, 31]
	*α*_2_	0.0 − 1.0	1 × 10^−8^	Cells day^−1^	[31]
	*a*_*I*_	0.001 − 1	0.03	Day^−1^	Estimated
	*b*_*I*_	0.001 − 1	0.03	Day^−1^	Assumed

*dM*/*dt*	*V*_*M*_	0 − 1	0.5	mg day^−1^	[18, 40]
	*d*_1_	0 − 0.1	0.011	Day^−1^	[18, 40]

*dR*/*dt*	*V*_*R*_	0 − 1	0.5	mg day^−1^	[19]
	*d*_2_	0 − 0.1	0.011	Day^−1^	[19]

## Data Availability

The data used to support the findings of this study are available from the corresponding author upon request.
